# Efficacy and safety of prophylactic balloon occlusion in the management of placenta accreta spectrum disorder: a retrospective cohort study

**DOI:** 10.1186/s12905-024-03049-4

**Published:** 2024-04-01

**Authors:** Kai Chen, Junyao Chen, Youliang Ma, Yanping Gan, Liyun Huang, Fang Yang, Yue Chen, Linlin Zhong, Sha Su, Yu Long

**Affiliations:** 1 Department of Obstetrics, The First Affiliated Hospital of Guangxi Medical University, Nanning, Guangxi Zhuang Autonomous Region China; 2 Department of Obstetrics and Gynecology, People’s Hospital Affiliated to Shandong First Medical University, Jinan, China

**Keywords:** Efficacy, Prophylactic balloon occlusion, Placenta accreta spectrum disorder, Risk, Safety

## Abstract

**Objective:**

Placenta accreta spectrum disorder (PAS) is a serious obstetric complication associated with significant maternal morbidity and mortality. Prophylactic balloon occlusion (PBO), as an intravascular interventional therapies, has emerged as a potential management strategy for controlling massive hemorrhage in patients with PAS. However, current evidence about the clinical application of PBO in PAS patients are still controversial. This study aimed to evaluate the effectiveness and safety of PBO in the management of PAS.

**Methods:**

A retrospective cohort study including PAS patients underwent cesarean delivery was conducted in a tertiary hospital from January 2015 to March 2022. Included PAS patients were further divided into balloon and control groups by whether PBO was performed. Groups were compared for demographic characteristics, intraoperative and postoperative parameters, maternal and neonatal outcomes, PBO-related complication and follow up outcomes. Additionally, multivariate-logistic regression analysis was performed to determine the definitive associations between PBO and risk of massive hemorrhage and hysterectomy.

**Results:**

A total of 285 PAS patients met the inclusion criteria were included, of which 57 PAS patients underwent PBO (PBO group) and 228 women performed cesarean section (CS) without PBO (control group). Irrespective of the differences of baseline characteristics between the two groups, PBO intervention did not reduce the blood loss, hysterectomy rate and postoperative hospital stay, but it prolonged the operation time and increased the cost of hospitalization (All *P* < 0.05) Additionally, there were no significant differences in postoperative complications, neonatal outcomes, and follow-up outcomes(All *P* > 0.05). In particular, patients undergoing PBO were more likely to develop the venous thrombosis postoperatively (*P* = 0.001). However, multivariate logistic regression analysis showed that PBO significantly decreased the risk of massive hemorrhage (OR 0.289, 95%CI:0.109–0.766, *P* = 0.013). The grade of PAS and MRI with S2 invasion were the significant risk factors affecting massive hemorrhage(OR:6.232 and OR:5.380, *P*<0.001).

**Conclusion:**

PBO has the potential to reduce massive hemorrhage in PAS patients undergoing CS. Obstetricians should, however, be aware of potential complications arising from the PBO. Additionally, MRI with S2 invasion and PAS grade will be useful to identify PAS patients who at high risk and may benefit from PBO. In brief, PBO seem to be a promising alternative for management of PAS, yet well-designed randomized controlled trials are needed to convincingly demonstrate its benefits and triage the necessity of PBO.

## Introduction

Placenta accreta spectrum disorder (PAS) is a life-threatening pregnancy complication associated with significant maternal morbidity and mortality [1]. In recent decades, the global prevalence of PAS has increased from 0.01 to 1.1% [2]. Unmanageable and catastrophic hemorrhage is the most common and serious complication of PAS since it may easily lead to hysterectomy, hemorrhagic shock, and even maternal-fetal death [3, 4]. PAS is strongly associated with the history of cesarean section (CS) [5]. Over the last 40 years, CS rates have increased rapidly from less than 10% to over 30%, indirectly leading to a nearly tenfold increase in the global incidence of PAS [6]. In China, the CS rate has risen gradually and maintained at a high level over the past three decades. With the full implementation of the “two-child” and “three-child” policies, the incidence of PAS is also dramatically rising, likely due to the rise in CS procedures, endouterine maneuvers, advanced maternal age, and the expanded use of assisted reproductive techniques [7, 8].

Despite significant advancements in the awareness and management of PAS, the rate of maternal mortality remains as high as approximately 7% [9, 10]. In the resource-limited and rural settings lacking excellent expertise and experience, approximately half of PAS patients require peripartum hysterectomy, which permanently affects fertility and causes great harm to patients’ physical and mental health [11]. Thus, PAS has become a global issue and major concern in obstetrics following its rapidly rising incidence, severe complications, and high healthcare costs [12]. Currently, the optimal management strategy for PAS remains highly challenging for obstetrician. Hence, there is a pressing need to explore the viable perioperative management and hemostasis methods, aiming to effectively control bleeding and preserve the uterus [13].

With the rapid development of interventional techniques, several prophylactic balloon occlusion (PBO) approaches, involving abdominal aorta(AA), common iliac artery(CIA), or internal iliac artery (IIA) occlusion, have been employed in the management of PAS [14, 15]. Theoretically, PBO can limit uterine perfusion during CS when indicated, thereby promoting the hemostasis intraoperatively [16]. This technique has shown promising outcomes in terms of reducing intraoperative blood loss and decreasing the likelihood of hysterectomy, but there are conflicting results regarding implementing PBO in PAS patients, ranging from affirmative to skeptical to inconclusive [1]. Meanwhile, PBO may also bring complications such as thrombosis diseases, haematoma and rarely artery rupture, and ischaemic necrosis of the lower limbs [17]. Despite accumulated studies conducted on balloon occlusion as a management strategy for PAS, current evidence remains of low quality and inconclusive owing to the limited literature and cases available. Also, the risks and benefits of PBO have not been well described, highlighting the need for further investigation to address the research gap in this field.

Moreover, China has a huge volume of PAS patients, but there is still a limited understanding about the optimal implementation of PBO [18]. The primary objective of this study is to evaluate the effectiveness and safety of PBO in the management of PAS and provide valuable insights for obstetrics when dealing with PAS patients. Specifically, we aim to assess its impact on maternal outcomes, neonatal outcomes, PBO-related complications, and long-term outcomes, etc. The findings from this research may contribute to the development of evidence-based guidelines and enhance clinical decision-making for the management of this complex and challenging obstetric condition.

## Methods

### Study design and data sources

A retrospective cohort study was conducted in the First Affiliated Hospital of Guangxi Medical University (a tertiary university hospital), which is a referral center for high-risk gravida and puerpera in South China. A multidisciplinary team (including senior obstetricians, anesthesiologists, gynecologic oncologists, interventional radiologists, vascular surgeons, neonatologists, urologists, ultrasound experts, intensive care units, and a well-stocked blood bank) for diagnosis and treatment of PAS is available in our institution. This study was approved by the institutional review board of our hospital (2015KY-E-042). Written informed consent was not required because of the retrospective nature of the review.

**Fig. 1:**
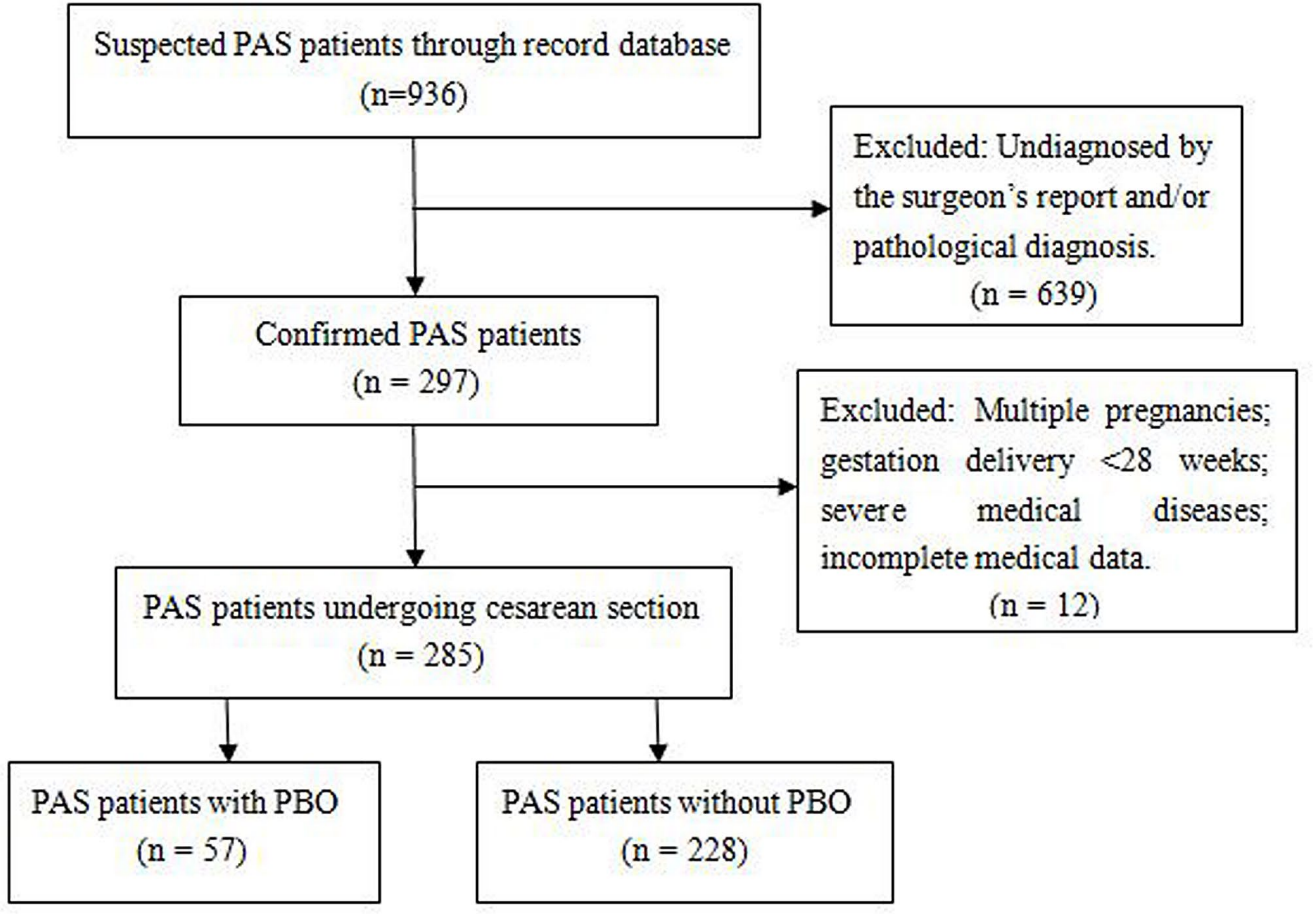
Flow chart of the patient’s enrollment and exclusion

Electronic medical record database of our institution was reviewed to identify available PAS cases undergoing CS from January 2015 to March 2022.The inclusion criteria were as follows: (1) PAS was detected by prenatal ultrasonography (US) or magnetic resonance imaging (MRI), and subsequently diagnosed by the surgeon’s intraoperative report and/or postoperative pathological diagnosis (Patients not diagnosed antenatal but were confirmed during the CS also be included); (2) Singleton pregnancy and gestation delivery ≥ 28 weeks; and (3) Elective or emergency CS. The following patients were excluded: multiple pregnancies; gestation delivery < 28 weeks; severe medical diseases (severe cardiac disease, severe dysfunction of liver and kidney, and abnormal coagulation function, etc.); aortic diseases; incomplete medical data. Finally, 285 PAS patients were included in this trial, the details were shown as a flowchart in Fig. 1.

### Data collection and follow-up

The following information were collected: maternal demographics (age, gravidity, parity, gestational age, BMI), past medical history, prior abortion and CS times, obstetric history, prenatal imaging findings, balloon occlusion procedure details, operative reports, intraoperative and postoperative complications, maternal and neonatal outcomes, and PBO-related complications. All patients without hysterectomy were followed up for a period of 6–18 months. US was performed to evaluate the uterine volume, and data on menstrual recurrence and changes in menstrual flow were obtained by follow-up telephonic interviews. MRI topography of the placental invasions was classified as area S1(above the peritoneal reflection, supplied by the uterine arteries) and area S2(below the peritoneal reflection, supplied by the collaterals of the internal pudendal arteries) [19]. According to the 2018 FIGO classification, the grade of PAS was classified into three subtypes: placenta accreta, increta, and percreta [20].

### PBO procedure and operative management

PBO procedures were implemented in a hybrid operating room with a C-arm for digital subtraction angiography (DSA) and performed by senior vascular surgeons under local anesthesia. The catheter was inserted via the right femoral artery, then the occlusion balloon was embedded and placed at the infrarenal abdominal aorta (48 cases), both common iliac arteries (7 cases) or internal iliac arteries (2 cases). The size of the balloon was chosen according to the diameter of the arteries (abdominal aorta, common iliac artery, or internal iliac artery) determined by preoperative US or MRI measurements.

CS was done under spinal anesthesia or general anesthesia. As soon as the fetus was delivered and the umbilical cord clamped, the occlusion balloon was inflated immediately to occlude the blood flow. Once satisfactory hemostasis was achieved, the balloon was slowly deflated. If bleeding was not controlled, the balloon would be deflated for 1 min every 10 min and further manipulation (such as uterine compression suturing, uterine artery ligation, intrauterine gauze packing, and intrauterine tamponade) was undertaken to establish hemostasis. Clinical application generally needed 1–3 cycles in our hospital. If all these methods did not successfully establish an ideal hemostasis, a total hysterectomy was performed. Surgical samples (including separated placentas, total or partial hysterectomy tissues, and partially resected myometrium with or without implanted placenta) were obtained whenever possible for histopathological confirmation. After the operation, the balloon catheter and sheath were immediately removed when observing no apparent vaginal bleeding. Then, the puncture sites were bandaged with pressure for at least 15 min and the lower limbs were immobilized for 2–4 h, so as to stabilize the initial hemostasis and the arterial access site [21, 22]. Based on the assessment of venous thromboembolism scores and the risk of postpartum hemorrhage, low molecular weight heparin were prophylactically administered to prevent thrombosis after 24 h postoperatively. In the control group, patients underwent CS without balloon occlusion, and perioperative management was similar in both groups except for the PBO procedure.

### Collection on outcomes of interest

Maternal outcomes and neonatal outcomes were collected. Maternal outcomes included estimated blood loss (EBL), massive hemorrhage (EBL ≥ 2000 mL)rate [23], hysterectomy, maternal death, operation time, urological injury, intraoperative hemostatic approaches, DIC, haemorrhagic shock, ICU admission, postoperative complications (e.g., puerperal infection, pneumonia, intestinal obstruction), PBO-related complications (including puncture site hematoma, thrombosis, embolic events, and vascular rupture), postoperative hospital stay, cost of hospitalization, and neonatal outcomes(neonatal weight, Apgar score, and neonatal asphyxia).The data regarding follow-up were as follows: gynecologic ultrasound, menstrual recovery time, menstrual volume, menstrual cycle, duration of menstruation, uterine involution, intrauterine adhesions, and cesarean scar diverticulum.

### Statistical analysis

All continuous variables were tested for normality using descriptive statistics for skewness and kurtosis, visual evaluation of histograms, and the Kolmogorov-Smirnov test. Quantitative variables were presented as the mean ± standard deviation (SD) or median and interquartile range (IQR) where appropriate, and qualitative variables were listed with frequency and percent. We used independent samples t test for normally distributed continuous variables and Mann-Whitney U test for nonnormally distributed continuous variables, respectively. Chi-square or Fisher’s exact test was applied to compare categorical variables, as appropriate. Additionally, multivariate-logistic regression analysis was performed to determine the association between PBO and risk of massive hemorrhage and hysterectomy, and adjusting for potential confounding variables including age, BMI, gravidity, parity, abortion, prior CS, surgery type (emergency CS or planned CS), MRI features, hemostasis manipulation, and PAS grading, etc. Odds ratios (ORs) and corresponding 95% confidence interval (CI) were calculated for binary outcomes. *P* < 0.05 was considered statistically significant. All analyses were performed using SPSS 24.0 software (IBM Corp, Armonk, NY, USA).

## Results

A total of 285 PAS patients underwent CS were included, in which 57 patients underwent PBO (PBO group) and 228 women performed without PBO (control group). Baseline characteristics are summarized in Table 1. Patients in both groups were similar in age, BMI, gravidity, gestational weeks, and abortion times, except for parity (*P =* 0.005) and number of previous CS (*P =* 0.001). The frequency of emergency CS was significantly lower in PBO group than in control group(*P =* 0.004). Higher proportion of patients in PBO group have an MRI with S2 invasion (71.2% vs. 28.6%, *P*<0.001).Moreover, more severe types (increta and percreta)of PAS were prevalent in the PBO group (96.5% vs. 61.0%, *P*<0.001).

**Table 1:** Baseline characteristics in subjects according to application of PBO

Variable	PBO group (*N* = 57)	Control group (*N* = 228)	*P*-value
Maternal Age (years )	33.58 ± 4.05	34.37 ± 4.42	0.498
BMI (kg/m^2^)	25.67 ± 2.94	26.80 ± 3.34	0.561
Gestational weeks (weeks)	36.4 ± 1.63	36.34 ± 2.22	0.079
Gravidity (number)	4 (3–4)	4 (3–4)	0.605
Parity (number)	2 (2–3)	2 (2–2)	0.005
Abortion(number)	1 (0–2)	1 (1–2)	0.254
Prior CS (number)	1 (1–1)	1 (0–1)	0.001
**Type of CS**			
Planed CS	50 (87.7%)	156 (68.4%)	0.004
Emergency CS	7(12.3%)	72 (31.6%)	
**Type of PAS**			< 0.001
Placenta Accreta	2 (3.5%)	89 (39.0%)	
Placenta Increta	23 (40.4%)	114 (50.0%)	
Placenta Percreta	32 (56.1%)	25 (11.0%)	
**MRI invasion topography**	*N* = 52	*N* = 210	
MRI with S1 invasion	15 (28.8%)	150 (71.4%)	< 0.001
MRI with S2 invasion	37 (71.2%)	60 (28.6%)	< 0.001

**Note**: The data were shown as median (interquartile range) or number (%)

**Abbreviations**: PBO, prophylactic balloon occlusion; BMI, Body mass index ;CS, cesarean section; PAS, Placenta accreta spectrum; MRI, Magnetic resonance imaging

Maternal and neonatal outcomes are summarized in Table 2. The mean EBL (1850mL vs. 950 mL, *P*<0.001) and operation time (186 min vs. 107 min, *P*<0.001) were obviously higher in the PBO group compared with control group. The frequency of massive hemorrhage(EBL ≥ 2000 mL)was also higher in the PBO group than in the control group (47.4% vs. 24.6%, *P* = 0.001). Hysterectomy rate and bladder injury rate were higher in the PBO group comparing with control group. The ICU admission rate in the PBO group (21.1% vs7.5%, *P* = 0.002) was significantly higher than that in the control group. The postoperative hospital stay of the PBO group was significantly longer than that of the control group (*P*<0.001), and the hospitalization cost was also higher than that of the control group (*P*<0.001). Other outcomes such as the incidence of puerperal infection, pneumonia, and intestinal obstruction did not differ between the two groups (*P* > 0.05). No maternal death was observed in either group.

**Table 2:** Maternal and neonatal outcomes in subjects according to application of PBO

Variable	PBO group (*N* = 57)	Control group(*N* = 228)	*P*-value
Operation time, min	186 (138–244)	108 (77–147)	< 0.001
EBL, ml	1800 (1000–3600)	1000 (500–1800)	< 0.001
EBL ≥ 2000 ml	27 (47.4%)	56 (24.6%)	0.001
Hysterectomy	13 (22.8%)	9 (3.9%)	< 0.001
Urological injury	8 (14.0%)	3 (1.3%)	< 0.001
ICU admission	12 (21.1%)	17 (7.5%)	0.002
Postoperative hospital length, days	8 (5.5-9)	5 (4–6)	< 0.001
Hospitalization cost, yuan	39,784 (33,303–48,179)	14,252 (11,248–19,442)	< 0.001
Puerperal infection	6 (10.5%)	13 (5.7%)	0.192
Pneumonia	1 (1.7%)	7 (3.1%)	0.591
Intestinal obstruction	4 (7.0%)	7 (3.1%)	0.166
Venous thrombosis of lower limb	6 (10.5%)	2 (0.8%)	0.001
Neonatal asphyxia	2 (3.5%)	6 (2.6%)	0.002
5-min APGAR score	9.5 (8–10)	10 (10–10)	0.72
Neonatal birth weight, g	2631.9 ± 447.8	2747.6 ± 555.4	0.165

**Note**: The data were shown as median (interquartile range) or number (%)

**Abbreviations**: EBL Estimated blood loss; ICU, Intensive care unit

PBO-related complications are also outlined in Table 2. In the PBO group, 6 (10.5%) patients developed venous thrombosis of lower limb, while there were only 2 cases in the control group (*P* = 0.077). Besides, no participants had other PBO-related complications, including puncture site hematoma, embolic events, and vessel dissection. As for neonates, there were no significant differences in neonatal weight and Apgar score (*P* = 0.165 and 0.72, respectively). However, the neonatal asphyxia rate was slightly lower in the PBO group (*P* = 0.002).

The follow-up outcomes were presented in Table 3. Of all patients included in the study, 263 women successfully preserved their uterus and were followed for 6-18months. Women in both groups reported a median time of 4 months and 3 months for menstruation renewal (*P* = 0.294). The hypomenorrhea and menstrual disorders after CS did not differ between the groups (*P* = 0.288 and *P* = 0.165, respectively). There were no differences with regard to the rate of cesarean scar diverticulum (*P* = 0.133) and intrauterine adhesion(*P* = 0.196) between the two groups.

**Table 3:** Obstetrical outcomes on follow up in subjects according to application of PBO

Variable	PBO group (*N* = 44)	Control group (*N* = 219)	*P*-value
Menstruation renewal, Months	4 (3–6)	3 (3–5)	0.294
Hypomenorrhea	4 (9.1%)	11 (5.0%)	0.288
Menstrual disorders	7 (15.9%)	19 (8.6%)	0.165
Cesarean scar diverticulum	3 (6.8%)	5 (2.3%)	0.133
Intrauterine adhesion	2 (4.5%)	3 (1.4%)	0.196

**Note**: The data were shown as median (interquartile range) or number (%)

Adjusted ORs of PBO for massive hemorrhage and hysterectomy were shown in Table 4. A multivariable logistic regression model was used to adjust for the relevant covariates that influence massive hemorrhage and hysterectomy. These covariates include age, BMI, gravidity, parity, prior abortion, prior cesarean section, severity of PAS, MRI features, CS type (emergency or planned), and other hemostasis manipulation. Multivariate regression analysis showed that PBO significantly decreased the risk of massive hemorrhage (adjusted OR 0.289, 95%CI:0.109–0.766, *P* = 0.013).In the multivariable regression model, the degree of PAS and MRI with S2 invasion were the significant risk factors for massive hemorrhage (*P*<0.001), while the remaining covariates didn’t exhibit any significance. After adjusting for the hysterectomy-related risk factors, multivariate analysis showed that PBO didn’t influence the hysterectomy rate (adjusted OR 2.189, 95% CI:0.568–8.430, *P* = 0.225). The grade of PAS was potentially associated with the increased risk of hysterectomy (*P* < 0.001),while other hemostasis manipulations were associated with a lower hysterectomy rate (*P* = 0.001).

**Table 4:** Adjusted odds ratios (OR) of PBO for massive hemorrhage and hysterectomy

Variable	Outcomes	Unadjusted OR		Multivariate-adjusted OR	
		OR (95% CI)	*P*-value	OR (95% CI)	*P*-value
	Massive hemorrhage				
PBO intervention		1.969(1.046–3.707)	0.036	0.289(0.109–0.766)	0.013
Grade of PAS				6.232(3.289–11.809)	< 0.001
MRI with S2				5.380(2.607–11.101)	< 0.001
	Hysterectomy				
PBO intervention		7.189(2.895–17.852)	< 0.001	2.189(0.568–8.430)	0.255
Grade of PAS				14.564(4.125–51.423)	< 0.001
Hemostasis manipulation				0.104(0.027–0.408)	0.001

Note: Multivariate-adjusted variables including age, BMI, gravidity, parity, abortion, prior cesarean section, surgery type, MRI features, hemostasis manipulation, and grade of PAS

## Discussion

### Principal findings

Present study showed that regardless of the differences of demographic and obstetric characteristics between the two groups, PBO did not reduce blood loss, nor did it reduce hysterectomy rate and postoperative hospital stay, but it prolonged the operation time and increased the cost of hospitalization in PAS patients. Additionally, there were no adverse effects on postoperative complications, neonatal, and follow-up outcomes with the implementation of PBO. In particular, patients undergoing PBO were more likely to develop the thrombosis postoperatively. However, multivariate logistic regression analysis showed that PBO significantly decreased the risk of massive hemorrhage.

### Results in the context of what is known

The modalities of PBO varied in many hospitals and published results are conflicting and do not allow the extrapolation of robust evidence on the actual role of PBO in improving the outcome of PAS [24]. For instance, a previous meta-analysis and some retrospective research reported that balloon occlusion of IIA had benefits in reducing blood loss, blood transfusion, and even the rate of hysterectomy in women with placenta accreta [17, 25–27]. Nevertheless, one recent study showed that PBO was associated with reduced blood loss only for patients who underwent AA balloon occlusion [28]. Although retrospective studies showed IIA occlusion was effective, one meta and 3 small sample RCTs did not find any benefit [1, 12, 29, 30]. Additionally, recent pooled data documented that AA occlusion was more effective in reducing intraoperative blood loss compared with IIA occlusion in PAS patients [10]. Our present study is partially in accordance with the existing literatures, which may be attributed to differences in the subjects, heterogeneity of PAS patients, research types and sample sizes, occlusion locations, and occlusion duration among these studies.

In the preliminary analysis, we didn’t find any benefit of PBO in terms of EBL, hysterectomy rate, and urological injury. However, the results should be interpreted critically, because the baseline characteristics were not fully balanced between the PBO group and control group. Specifically, patients in the PBO group had a greater number of CS history, MRI sign with S2 invasion, and higher proportion of PAS grade 2/3. Indeed, PAS patients with multiple CS and severe PAS grade often presented with pelvic adherences, a thin and hypervascular lower uterine segment, extensive collateral circulation, and greater extent of placental invasion, as well as excessive invasion to bladder, cervix, and parametrium [31, 32]. Therefore, massive bleeding is more likely to occur in these PAS patients, increasing the surgical difficulties and urologic injuries [30, 33, 34]. Even with the support of PBO, it remains challenging to avoid massive hemorrhage and bladder injuries, and prejudge the chances of preserving the uterus. In this context, while our initial results did not support the improvement of aforementioned outcomes, it’s still logical and promising within the current constraints. We reckon that PBO may exhibit significant benefits when applied to appropriately matched subjects. As expected, our multivariate result supports a significant benefit of PBO in decreasing the risk of massive hemorrhage. Admittedly, PBO provides a cleaner operating field for surgeons to perform surgical procedure, accordingly decreasing the complexity of the operation. With the assistance of PBO, obstetricians can quickly remove the implanted placental tissue, easily perform compression hemostatic sutures, and proceed accurately with bladder dissection, indirectly reducing the intraoperative hemorrhage and operative time [35].

The safety of PBO procedures is also a significant concern. PBO-related complications are the most important features need to focus on, including initial vessel injury, arterial thrombosis, puncture point haematoma, ischaemic necrosis of the lower limbs, and rarely arterial rupture [36]. Thrombosis is one of the most common complications of PBO, with a reported incidence ranging from 5–15% [34, 35]. A systematic review reported that the overall incidence of PBO-related complications was 5%, while one study did not show any serious PBO-related complications occurred in the balloon group [15, 17]. In our study, we only found 10.5% of cases experienced venous thrombosis of lower limb in the PBO group, without any other PBO-related complications. Possible reasons for venous thrombosis may mainly be attributed to the slow blood flow arising from the balloon occlusion. However, other factors such as hypercoagulable state, severe blood loss, unstable hemodynamics, blood transfusion, and secondary coagulation dysfunction might also contribute to postoperative thrombosis. In our study, AA occlusion (84.2%) was the main approach of PBO. As compared with the occlusion of IIA or the CIA, AA occlusion reduce the complexity of procedure and conserve the operative time and block most of the collateral vessels, which in turn improve the occlusion effect and decreases the risk of related complications and radiation exposure [37, 38]. It was reported that selecting the appropriate balloon size, involving experienced interventional radiologists familiar with balloon occlusion techniques, and meticulously controlling the occlusion time are crucial factors for reducing the risk of complications associated with PBO [36, 39]. In our study, the diameter of occlusion arteries was measured before surgery to determine the appropriate size of the balloon catheter. Besides, the balloon was inflated for 10 min at a time with an interval of 1 min during surgery, possibly reducing the ischemic time. Additionally, the PBO procedure in a hybrid operating room is useful to eliminate the potential risk of balloon catheter displacement [8]. Clinically, the adverse complications should be minimized, and PAS patients should be objectively informed of the potential complication risk of PBO.

For neonatal outcomes, current results showed no difference between the PBO group and control group, reinforcing its safety profile for neonates. Additionally, a controversial issue is whether PBO radiation exposure will cause fetal damage. In our study, balloon insertion was performed rapidly by an experienced radiologist. The fetal radiation exposure dose was under 10 m Gy, far less than the standard dose of ≤ 100 mGy recommended by the International Commission on Radiological Protection (ICRP) [40]. Also, we explored previously uninvestigated concepts about the long-term effect of PBO intervention for PAS patients. Generally, the bloodstream occlusion can induce ischemic injury to the ovaries and uterus, potentially leading to ovarian dysfunction, abnormal uterine involution and menstrual disorders. Through follow-up, we found no significant difference in menstrual recovery, menstrual disorders, cesarean scar diverticulum, and intrauterine adhesion between the 2 groups, indicating that PBO had no long-term adverse effects for ovary or uterus. Based on these findings and current literatures, we believe that with the advance of PBO technique, it remains a promising procedure applying for the management of PAS.

### Clinical implications

Currently, the definite efficacy of PBO in patients with PAS is yet to be confirmed, and our findings will enrich the available data and expand our understanding in this field. In our study, we highlight the potential of applying PBO in PAS patients for reducing the massive hemorrhage, possibly transferring to a lower risk of DIC, haemorrhagic shock and hysterectomy. In addition, MRI with S2 invasion and higher PAS grade were proved to be capable of predicting the massive hemorrhage risk, which will be useful to identify PAS patients who are at high risk and may benefit more from PBO. Accurate prenatal diagnosis of PAS is a requisite for the implementation of PBO in clinical practice. Prenatal diagnosis of PAS is commonly evaluated by US while prenatal MRI is widely employed to diagnose and describe the depth and topography of placental invasion [36]. Therefore, accurate depiction of invasion topography for PAS patients using MRI would facilitate preoperative planning and evaluation of implementation of PBO. Additional methods to more precisely evaluate the severity of PAS prior to CS are critical for optimizing PBO application. Interestingly, one encouraging study recommended that the “intraoperative staging” of PAS allows for the optimization of the use of endovascular balloon occlusion and decreases its frequency of use without increasing the volume of blood loss [41]. Thus, to structure a tailored prediction models integrating our finding and previous classical characteristics may improve the ability to define the PBO inclusion criteria prenatally.

### Strengths and limitations

Some strengths should be pointed out in the present study. First, our study had a relatively large sample size and included abundant adjusting factors, with adequate power to detect differences for main outcome. Second, our trial had strict inclusion criteria and PAS diagnosis was made clinically according to surgeon’s intraoperative report and confirmed by detailed histopathology wherever possible, so it’s better to accurately distinguish the subtypes of PAS. Third, we carefully appraise the maternal and neonatal outcomes. While our study provides valuable insights, several limitations should be acknowledged. Firstly, the reliance on medical records for data collection introduces the possibility of missing information. Secondly, this study was conducted at a single institution, limiting the generalizability of our findings. Thirdly, the imbalance in baseline characteristics may partially affect our analysis for the initial results. Finally, the study design was retrospective, which may introduce inherent biases and confounding factors.

### Research implications

Despite these limitations, our study further advances our understanding and optimizes the implementation of PBO in the clinical management of PAS, but future research should address several key areas. Firstly, prospective RCTs with larger sample sizes are needed to establish the efficacy and safety of this technique definitively. Follow-up studies are also necessary to evaluate the reproductive outcomes and long-term sequelae for both mothers and their children. Moreover, further investigation into the optimal timing, duration, and technique of balloon occlusion is warranted to optimize the protocol. Lastly, exploring the cost-effectiveness and feasibility of implementing PBO in different healthcare settings would provide diverse information for healthcare decision-makers. However, it should be emphasized that when applying PBO, the severity of PAS, level of local medical care, and willingness of patients to retain fertility should be taken into consideration.

## Conclusions

In conclusion, PBO can be considered as a promising approach in the multidisciplinary management of PAS, particularly in the subset of patients with MRI indicating S2 invasion or severe PAS grade. However, the benefits of PBO must be balanced with the risk of this procedure, and indications should be strictly controlled. Furthermore, well-designed RCTs are urgently needed to triage the necessity of PBO and to establish whether its benefits may outweigh its potential adverse effects to justify its implementation.

## Data Availability

The datasets generated and analysed during the current study are not publicly available due to privacy protection but are available from the corresponding author on reasonable request.

## Abbreviations

PAS: Placenta accreta spectrum disorder
CS: Cesarean section
AA: Abdominal aorta
CIA: Common iliac artery
IIA: Internal iliac artery
PBO: Prophylactic balloon occlusion
US: Ultrasonography
MRI: Magnetic resonance imaging
DSA: Digital subtraction angiography
DIC: Disseminated intravascular coagulation
ICU: Intensive Care Unit
EBL: Estimated blood loss
IQR: Interquartile range
OR: Odds Ratio
CI: Confidence interval
